# Anti-PD-1 Therapy Enhances the Efficacy of CD30-Directed Chimeric Antigen Receptor T Cell Therapy in Patients With Relapsed/Refractory CD30+ Lymphoma

**DOI:** 10.3389/fimmu.2022.858021

**Published:** 2022-04-01

**Authors:** Wei Sang, Xiangmin Wang, Hongzhi Geng, Tianci Li, Dashan Li, Bingpei Zhang, Yi Zhou, Xuguang Song, Cai Sun, Dongmei Yan, Depeng Li, Zhenyu Li, Caixia Li, Kailin Xu

**Affiliations:** ^1^ Department of Hematology, The Affiliated Hospital of Xuzhou Medical University, Xuzhou, China; ^2^ Blood Diseases Institute, Xuzhou Medical University, Xuzhou, China; ^3^ Key Laboratory of Bone Marrow Stem Cell, Xuzhou, China; ^4^ Department of Hematology, The First Affiliated Hospital of Soochow University, Suzhou, China

**Keywords:** PD-1, chimeric antigen receptor, CD30, efficacy, relapsed/refractory CD30+ lymphoma

## Abstract

Anti-CD30 CAR-T is a potent candidate therapy for relapsed/refractory (r/r) CD30+ lymphomas with therapy limitations, and the efficacy needed to be further improved. Herein a multi-center phase II clinical trial (NCT03196830) of anti-CD30 CAR-T treatment combined with PD-1 inhibitor in r/r CD30+ lymphoma was conducted. After a lymphocyte-depleting chemotherapy with fludarabine and cyclophosphamide, 4 patients in cohort 1 and 3 patients in cohort 2 received 10^6^/kg and 10^7^/kg CAR-T cells, respectively, and 5 patients in cohort 3 received 10^7^/kg CAR-T cells combined with anti-PD-1 antibody. The safety and the efficacy of CAR-T cell therapy were analyzed. Cytokine release syndrome (CRS) was observed in 4 of 12 patients, and only 1 patient (patient 9) experienced grade 3 CRS and was treated with glucocorticoid and tocilizumab. No CAR-T-related encephalopathy syndrome was observed. Only two patients in cohorts 2 and 3 experienced obviously high plasma levels of IL-6 and ferritin after CD30 CAR-T cell infusion. The overall response rate (ORR) was 91.7% (11/12), with 6 patients achieving complete remission (CR) (50%). In cohorts 1 and 2, 6 patients got a response (85.7%), with 2 patients achieving CR (28.6%). In cohort 3, 100% ORR and 80% CR were obtained in 5 patients without ≥3 grade CRS. With a median follow-up of 21.5 months (range: 3_-_50 months), the progression-free survival and the overall survival rates were 45 and 70%, respectively. Of the 11 patients who got a response after CAR-T therapy, 7 patients (63.6%) maintained their response until the end of follow-up. Three patients died last because of disease progression. Taken together, the combination of anti-PD-1 antibody showed an enhancement effect on CD30 CAR-T therapy in r/r CD30+ lymphoma patients with minimal toxicities.

## Introduction

Chimeric antigen receptor (CAR) T-cell therapy offers an effective therapeutic option for patients with lymphoid malignancies (1–3). CD19 antigen was commonly selected in CAR-T treatment of B cell hematologic malignancies and got a response of 80% overall response rate (ORR) in lymphomas (2, 4) and higher than 90% ORR in B acute lymphocytic leukemia (B-ALL) patients (5) with controllable toxicities. However, most patients do not have a durable response, and there remains a room for improvement (6). The use of combinatorial approaches, including immunomodulatory drugs, checkpoint inhibitors, BTK inhibitor, *etc.*, have been tried in CAR-T cell therapy and showed a synergetic anti-tumor effect with endurable toxicities (7).

CD30 is a target universally expressed in virtually all classical Hodgkin lymphomas (cHL), anaplastic large cell lymphomas (ALCL), and in a proportion of other lymphoma types (8). Intensive chemotherapy followed by hematopoietic stem cell transplantation (HSCT) is commonly used in those r/r lymphoma patients, but a considerable proportion of patients eventually relapse after treatment (9). The CD30-specific antibody drug conjugate brentuximab vedotin treatment is an important immunotherapy option at present, with promising anti-tumor activity and manageable toxicity in cHL and CD30-positive peripheral T-cell lymphoma (10), but the long-term disease control capacity really needed to be improved. More than half of patients will eventually relapse (11), so it is imperative to develop novel effective therapeutics to improve the prognosis for patients of those lymphoma subtypes.

CD30-directed CAR-T cell therapy offers a remarkable opportunity to these r/r CD30+ lymphoma patients. However, although well tolerated, the anti-tumor activity of CD30 CAR-Ts in r/r cHL or ALCL needed to be further improved (12, 13). In a previous phase I study, the anti-CD30 CART cell infusion just yielded a 39% ORR (12). Strategies to enhance the curative effect of CD30 CAR-T cells, including lymphodepleting chemotherapy regimen optimization (14) and combination of PD-1 inhibitor (15), have emerged as new foci on research in recent years. PD-1 was shown to be upregulated by nearly 40% in activated CAR-T cells (16). Besides this, following activation, CAR-T cells could upregulate programmed death ligand-1 (PD-L1) expression on cancer cells, which leads to the lack of clinical efficacy of CAR-T cells (17). Furthermore, PD-1 blockade was shown to improve the expansion and persistence of CAR-T cells through interrupting the PD-1/PD-L1 pathway (18). Based on the critical role of the PD-1/PD-L1 axis in the anti-CD30 CAR-T cell therapies, the combination treatment with PD-1-blocking antibody has become a work worthy of exploration in the future.

Herein, in this study, we performed a combinatorial strategy with anti-PD-1 and anti-CD30 CAR-T cell treatment in patients with r/r CD30+ lymphomas and showed a synergetic anti-tumor activity of immune checkpoint inhibitor with minimal toxicities.

## Patients and Methods

### Patients

A multi-center phase II clinical trial (ClinicalTrials.gov identififiers: NCT03196830) was conducted in the Affiliated Hospital of Xuzhou Medical University and the First Affiliated Hospital of Soochow University. The inclusion criteria were as follows: (1), All the recruited relapsed or refractory lymphoma patients in this study must be confirmed CD30-positive through immunohistochemistry staining by at least two pathologists (2), Eastern Cooperative Oncology Group performance status of 2 or less (3), have ≥1 cm of measurable lesion, and (4) experienced disease progression after at least 1 line of systemic chemotherapy regimen concluded at least 1 month prior. The exclusion criteria include (1) severe organ dysfunction (2), a history of active systemic autoimmune or immunodeficiency disease, and (3) treatment history of immunosuppressive agents or glucocorticoids within the last month. All patients provided written informed consent in accordance with the Declaration of Helsinki before enrolling in the study. The study protocol and the consent forms were approved by the ethics committee of the Affiliated Hospital of Xuzhou Medical University and the First Affiliated Hospital of Soochow University.

### Treatment Protocol

**Figure 1:**
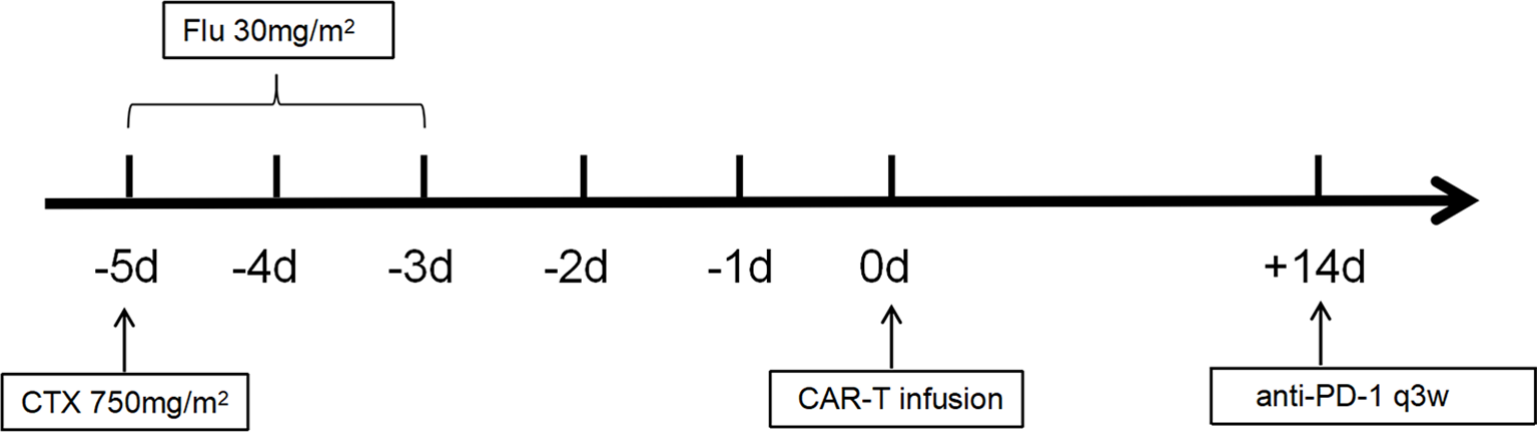
Flowchart for anti-CD30 CAR-T trials.

CD30 CAR-Ts were generated *via* a lentiviral vector. After a lymphocyte-depleting chemotherapy with (FC regimen (three daily doses of fludarabine, 30 mg/m², -5–3 days before infusion; one dose of cyclophosphamide, 750 mg/m², -5 days), the patients received a single dose of autologous CD30 CAR-T cell infusion intravenously (**Figure 1**).

The patients enrolled in this study were divided into 3 cohorts. The patients of cohort 1 and cohort 2 received 10^6^/kg and 10^7^/kg of CAR-T cells, respectively. The patients of cohort 3 received additional anti-PD-1 antibody at 14 days after 10^7^/kg CAR-T cell infusion, and this continued every 3 weeks (**Figure 1**). Sequential auto-HSCT was done in part of patients. A second infusion of CD30 CAR-Ts was allowed in patients who had progressive disease (PD) after the first treatment or after auto-HSCT.

### Outcomes

The priority of this study was to assess the safety and feasibility of CD30 CAR-Ts. Cytokine release syndrome (CRS) was graded according to the ASBMT consensus (19). Grade 3 or higher CRS was considered to be severe. Neurotoxic effects were assessed according to the National Cancer Institute Common Terminology Criteria for Adverse Events, version 4.03. The anti-tumor activity of anti-CD30 CAR-T cells was assessed and shown as ORR, complete remission (CR), partial remission (PR), overall survival (OS), and progression-free survival (PFS). OS was defined as the time from infusion to the date of death from any cause. PFS was defined as days from CD30 CAR-T cell infusion to relapse, progression, or death.

### Statistical Analysis

The sample size was based on clinical considerations. This is an exploratory study, and all analyses are descriptive in nature. Frequencies or percentages for categorical variables were used to analyze the safety and efficacy of CAR-T therapy. Follow-up time was estimated using the Kaplan–Meier method, whereas OS and PFS were estimated using the Kaplan–Meier method. Data were analyzed using Graphpad Prism version 8.

## Results

### Patient Characteristics

**Figure 2:**
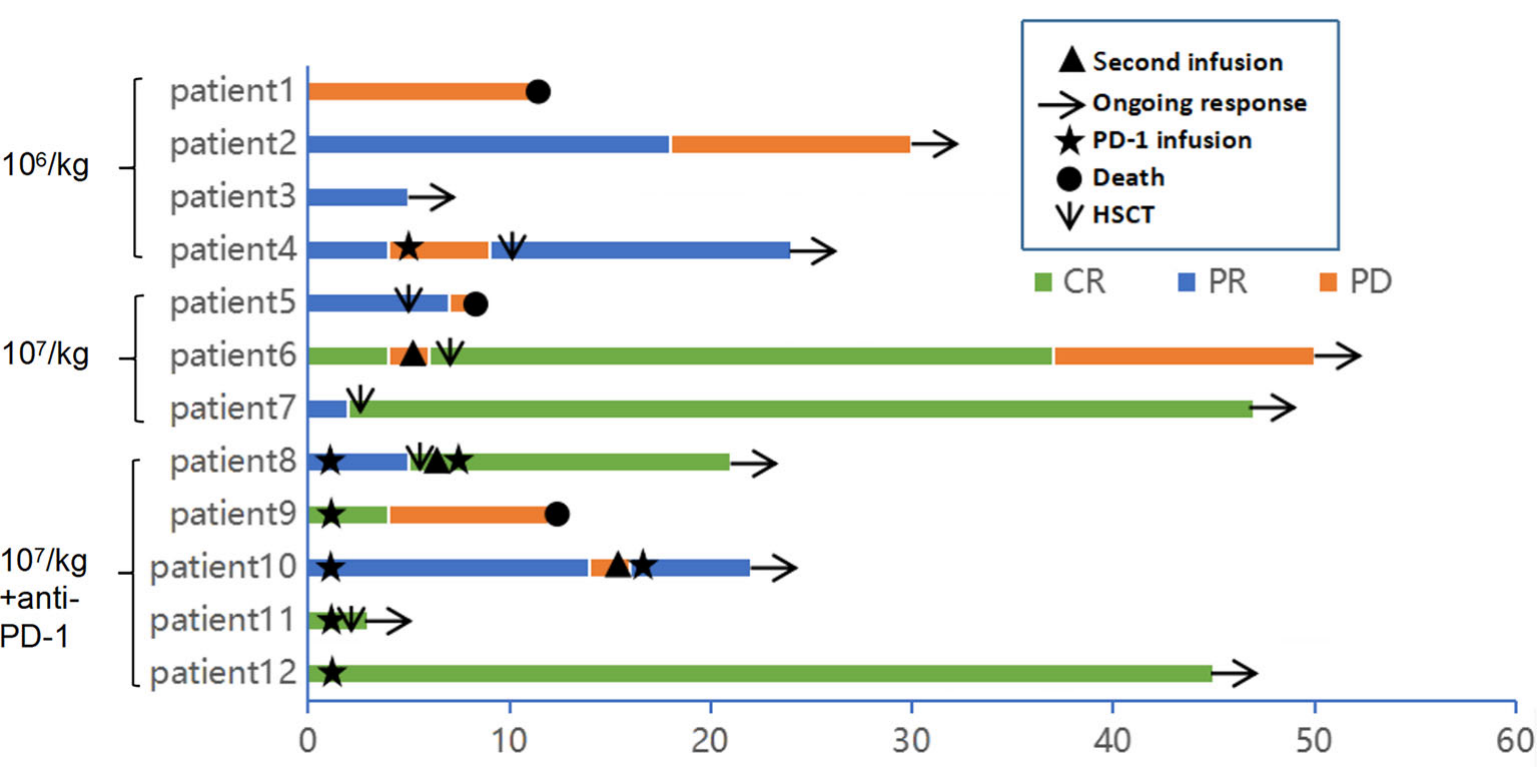
Clinical response and duration for patients after anti-CD30 CAR-T cell infusion. The color and the length of each bar indicate the response to the anti-CD30 CART treatment and the duration of response, respectively. CR, complete remission; PR, partial remission; PD, progressive disease. The black triangle indicates the start time of the second cycle of CAR-T cell infusion. The arrow indicates a sustained response. The star indicates the start time of anti-PD-1 antibody therapy. The original point and the downward pointing arrow represent the death and the start time of hematopoietic stem cell transplantation.

From July 1, 2017 to July 31, 2021, 13 patients including 9 cHL, 2 angioimmunoblastic T-cell lymphoma (AITL), and 2 gray zone lymphoma patients were enrolled. Finally, a total of 12 patients were evaluated for response because 1 AITL patient was lost to follow-up. The patient characteristics are summarized in **Table 1**. The median age was 25 years old (range: 19–64 years), and 7 patients were male patients. Most of them have complex pretreatment histories, including chemotherapy, radiotherapy, anti-PD-1 antibody, or even HSCT. At the time of enrollment into the CD30 CAR-T cell protocol, 9 of the 12 patients have extranodal invasion, including lung, bone, liver, muscle, *etc.* The disease status of all patients was PD before CD30 CAR-T infusion. Three patients received two cycles of CD30 CAR-T treatment. All the patients received FC lympho-depleting chemotherapy prior to CD30 CAR-T infusion. Sequential auto-HSCT and allo-HSCT were conducted in 5 and 1 patient, respectively. In total, in cohort 1, 4 patients received 10^6^/kg CAR-T cells; in cohort 2, 3 patients received 10^7^/kg CAR-T cells; and in cohort 3, 5 patients received 10^7^/kg CAR-T cells combined with anti-PD-1 antibody (**Figure 2**).

**Table 1:** Baseline characteristics of patients.

Patientnumber	Age(years)	SeX	Diagnosis/stage	Status of pre-CAR-T	Extranodal sites	Chemotherapy regimens	RT	ASCT	CAR-T infuse X1	Bridge anti- PD-1	CRSgrade	Best response	Current status
									06fkg				
	21	M	HLIIIIA	PD	Lung	ABVD*4, GDP*2, BGB-A317, PD-1*8	N	N	8.5	N	0	NR	Death
2	19	M	HLIIVB	PD	Bone, spleen	ABVD*6	N	N	7.6	N	0	PR	Relapsed
3	25	M	HLIIIIB	PD	N	ABVD*6, PD-1+ABVD*4, ABVD*1, RT*1	y	N	6.2	N	0	PR	PR
4	27	M	GZLIIIB	PD	N	R-EPOCH*6, R-ESHAP*4, PD-	N	y	5	N		PR	PR
						1*1							
5	27	F	GZLI	PD	Lung, mediastinal mass	R-CHOP*4, ABVD*4, GDP*2	N	Allo-HSCT	25	N	3	PR	Death
			IVB										
6	52	F	AITLIIV	PD	N	CHOP*8, DICE+ chidamide*2	N	Y	15.4/10.2	N	111	CR	Relapsed
			B										
7	29	F	HLIIVB	PD	Bone marrow,	ABVD*6,1GEV*6, HD-MTX*1	N	Y	12.6	N	0	CR	CR
					adnexa uteri								
8	24	M	HLIIVB	PD	Liver, muscle, bone	VAMP*2, COPDAC*3, ABVD*2, EDAP*1,	y	Y	12.9/14.8	Y	0/0	CR	CR
						COPDAC*1, GDP*2,							
						Gemcitabine+VP-16*1,							
						GEMOX*1, GDP*1, PD-1 *16,							
						RT, DHAP + lenalidomide							
9	64	F	HLIIVB	PD	Bone	CHOP*1, CHOPE*6, GCD*4,	y	N	14.2/12.6	Y	2	CR	Death
						ICE*4, PD-1*13, lenalidomide							
10	25	M	HLIIVA	PD	Bone,	*2, RT*15 Chemo *3, ABVD*5, PDL-	N	N	12.2	Y	0/0	PR	PR
					nasopharynx	1*4, PD-1*7							
11	19	F	HLIIVB	PD	Bone, lung	ABVD*2, PD-1+AVD·*4	N	Y	13.6	Y	0	CR	CR
12	19	M	HLIIVB	PD	Mediastinal mass	ABVD*6, RT, ESHAP*3, PD-1*4, ASCT*1	y	N	20	Y	0	CR	CR

M, male; F, female; HL, Hodgkin lymphoma; GZL, gray zone lymphoma; AITL, angioimmunoblastic T-cell lymphoma; RT, radiation therapy; ASCT, autologous stem cell transplantation; CRS, cytokine release syndrome; PD, progressive disease; CR, complete remission; PR, partial remission; NR, no response.

### Safety

**Figure 3:**
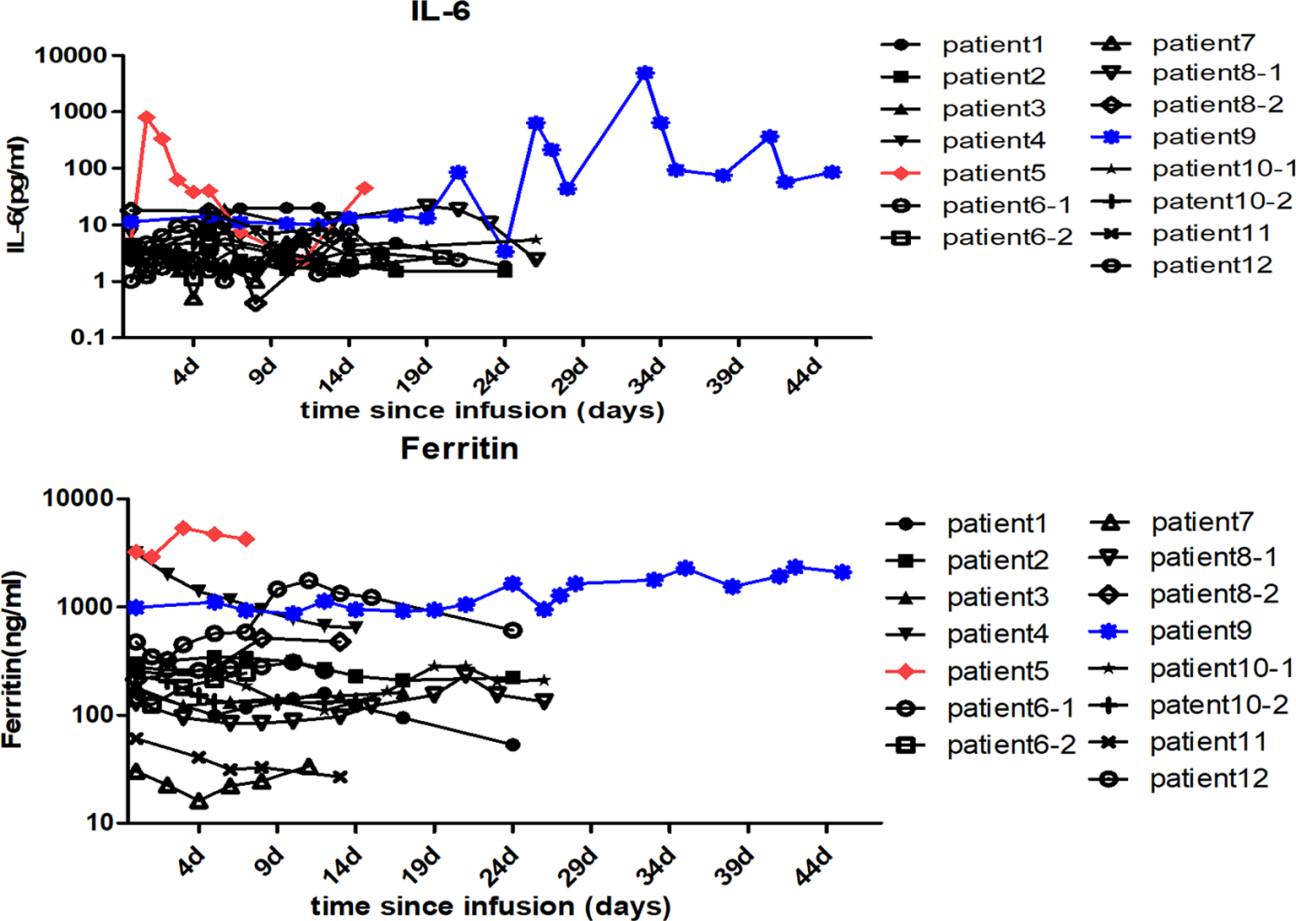
Changes in the patients’ serum cytokine levels after anti-CD30 CAR-T cell infusion. The serum IL-6 and ferritin levels of each patient were assessed before and at serial time points after anti-CD30 CAR-T cell infusion. The red and the blue lines, respectively, represent patient 5 and patient 9 with higher IL-6 and ferritin levels than the others.

The CD30 CAR-T infusions were well tolerated, and no treatment-related deaths occurred during the study. CRS was the most common non-hematological adverse event after CD30 CAR-T infusion. In total, 4 patients (33.3%) experienced CRS, and severe CRS occurred in one patient (patient 5). Fever occurred in all the 4 CRS patients, hypoxia occurred in 1 patient (patient 9), and hypotension occurred in 1 patient (patient 5) (**Table 2**). Only patient 9 was treated with glucocorticoid and tocilizumab. Patient 5 experienced obviously elevated plasma levels of IL-6 and ferritin immediately after CD30 CAR-T cell infusion. Intriguingly, delayed IL-6 peak occurred at 1 month after cell infusion in patient 9 and was sustained for 1 week (**Figure 3**). Six patients (50%) experienced cytopenias, including neutropenia, thrombocytopenia, or anemia, among which grade 3 or higher hematological toxicities happened in 5 cases (41.7%). Transient elevation of ALT/AST was observed in 3 patients (25%). Fatigue, nausea/vomiting, diarrhea, and skin rash were observed in 4 patients (33.3%), 2 patients (16.7%), 1 patient (8.3%), and 1 patient (8.3%), respectively. No neurotoxicity was observed during this study (**Table 2**).

**Table 2:** Adverse events of patients after anti-CD30 CAR-T cell infusion.

	Number of patients (%, *n* = 12)	
Adverse events	All grades	Grades 2–3
CRS	4 (33.3)	1 (8.3)
Fever	4 (33.3)	
Hypoxia	1 (8.3)	
Hypotension	1 (8.3)	
ICANS	0 (0)	0 (0)
Fatigue	4 (33.3)	2 (16.7)
Nausea/vomit	2 (16.7)	0 (0)
Diarrhea	1 (8.3)	0 (0)
Hematologic events	6 (50)	5 (41.7)
ALT/AST elevation	3 (25.0)	2 (16.7)
Rash	1 (8.3)	0 (0)

CRS, cytokine release syndrome; ICANS, immune effector cell-associated neurotoxicity syndrome.

### Efficacy

**Figure 4:**
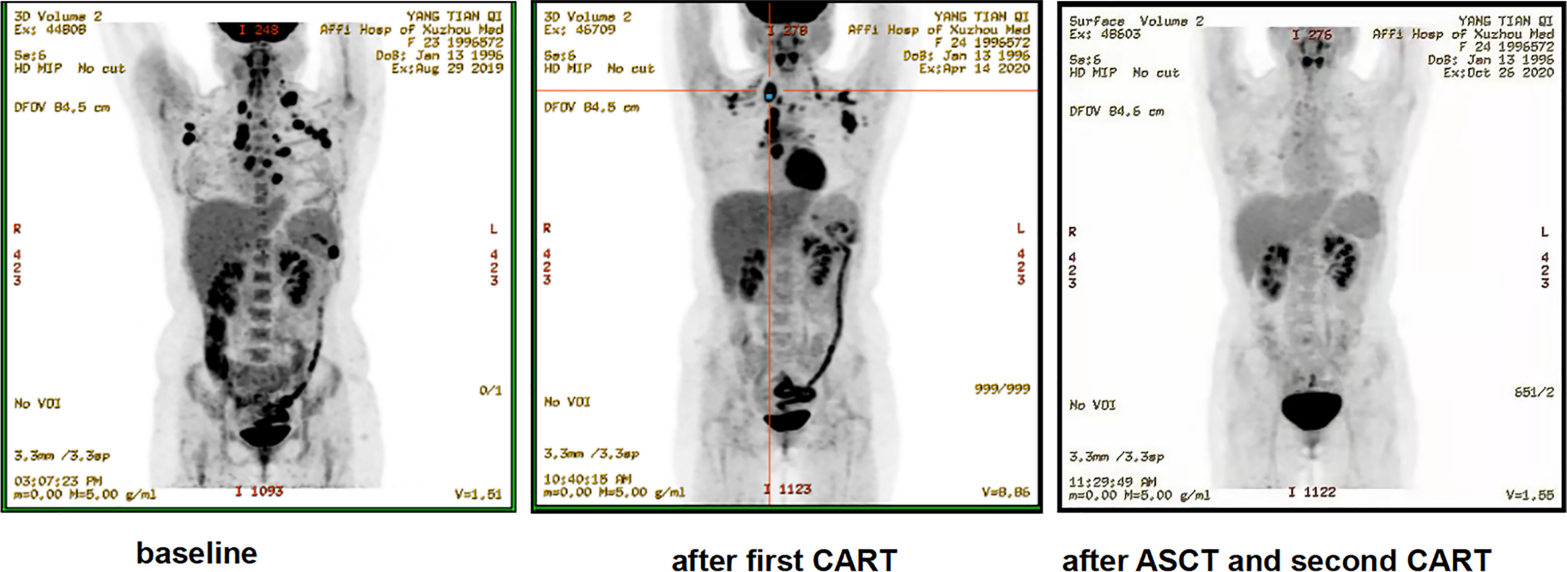
Representative clinical response images of the patient after anti-CD30 CAR-T cell infusion (patient 8). The second cycle of anti-CD30 CAR-T cells was infused 2 weeks after the autologous stem cell transplantation (ASCT). Positron emission tomography–computed tomography scans demonstrated a significant shrinkage of masses after the first cycle of anti-CD30 CAR-T cell infusion and complete disappearance of abnormal lymph nodes after ASCT and the second cycle of anti-CD30 CAR-T treatment.

**Figure 5:**
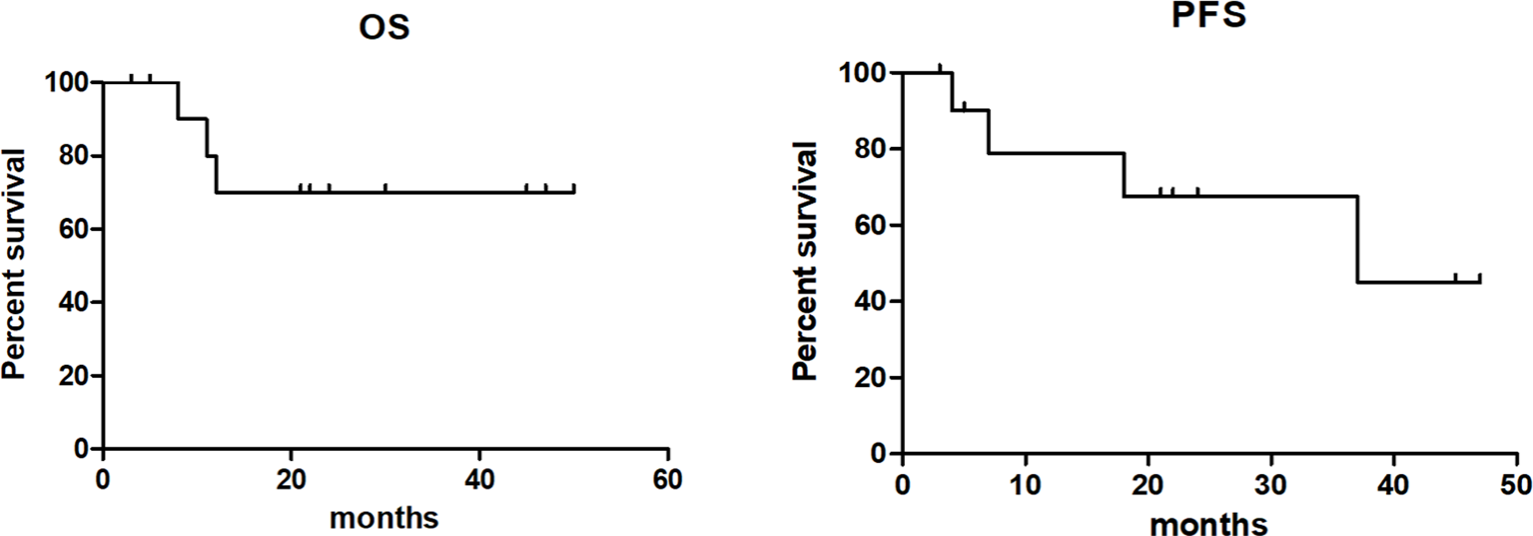
Overall survival and progression-free survival of patients after anti-CD30 CAR-T cell infusion.

Of the 12 evaluable patients, 6 patients had CR and 5 patients had PR after infusion, with 91.7% ORR and 50% CR rate (**Figure 2**, **Table 1**). In cohort 1, 3 of 4 patients achieved PR. In cohort 2, 2 of 3 patients achieved CR and 1 achieved PR, while in cohort 3, 100% ORR and 80% CR rate were achieved. Among 3 patients receiving a second CAR-T cell infusion, 2 patients achieved CR and 1 patient achieved PR. In the 11 patients responding to CAR-T treatment, 7 patients (7/11) continued to have responses until October 31, 2021. Of the 6 patients achieving CR, 4 patients continued to sustain a CR status until now. Among the 9 patients with cHL, 8 patients had response and 5 patients achieved CR (55.6%). Five of six (83.3%) cHL patients receiving 10^7^/kg CAR-T cells ± PD-1-blocking antibody achieved CR, while no CR was obtained in patients receiving 10^6^/kg CAR-T cells (cohort 1). Of 5 patients receiving HSCT after CAR-T cell infusion, 4 had CR and 1 had PR, with 100% ORR and 80% CR rate (**Figure 2**, **Table 1**). In particular, patient 8 in cohort 3, who received auto-HSCT and secondary CD30 CAR-T treatment immediately after achieving PR of the first CD30 CAR-Ts infusion, finally obtained CR and sustained remission until now (**Figure 4**). At a median follow-up of 21.5 months (range: 3–50 months), the PFS and OS were 45 and 70%, respectively (**Figure 5**). In total, 3 patients died lastly because of PD. Patient 1 (cohort 1) did not benefit from CAR-T and died 11 months after cell infusion. Patient 5 (cohort 2) and patient 9 (cohort 3) had PR and CR and died at 8 and 12 months post-CAR-T cell infusion, respectively.

## Discussion

CD30 CAR-T is an important treatment option for r/r CD30+ lymphoma, but the efficacy requires further improvement. In our multi-center study, based on the strategy of combinatorial treatment, anti-PD-1 antibody combined with CD30 CAR-T achieved encouraging results of 100% ORR and 80% CR rate with minimal toxicities.

For CD19 CAR-T, nearly 1/3 B-ALL patients and up to 13% of B-cell lymphoma patients were shown to undergo grade 3 or higher CRS (20, 21). While CAR-T targeting CD30 was shown to be well tolerated clinically, with rare cases of CRS and neurological toxicity (12, 13). In this study, based on the dosage explorations of CAR-T cells and the combinatorial strategy of PD-1 blockade, 3 cohorts of patients were conducted. In total, 8.3% severe CRS reaction was observed, and no neurotoxicity was found, and only 2 patients had significantly high plasma levels of IL-6 and ferritin after CAR-T treatment, which was consistent with the previous CD30 CAR-T studies of low toxicity characteristics (12, 13). Notably, our study showed that both 10^7^/kg dose level of CAR-Ts and the combinatorial treatment of anti-PD-1 antibody were safe with acceptable toxicities. The most probable related adverse event was hematologic events, which happened in about 50% of patients and may be partly attributed to lymphodepletion. Severe cytopenia was common (41.7%) but recoverable with proper management. Other toxicities, including nausea, rash, and diarrhea, were transient and endurable in our study.

It was hard for CD30 CAR-Ts alone to get a satisfactory clinical response in the previous studies (12, 13). The strategy of combining with PD-1 inhibitor to refuel the CAR-T cells was being tried, and this obtained a gratifying initial result (18). In our study of evaluable patients, CD30 CAR-Ts have a significant clinical activity in heavily pretreated r/r CD30+ lymphoma patients with ORR of 91.7% and CR rate of 50%. However, the clinical efficacy of 10^6^/kg CAR-T is not exciting because no patient obtained CR in cohort 1. In cohorts 2 and 3, a high dose of CAR-T and a combinatorial strategy of anti-PD-1 antibody significantly improved the therapeutic response of CAR-T, especially in cohort 3, of which 80% CR and 100% ORR was achieved. Furthermore, bridging HSCT after CAR-T treatment was done in 6 patients and got responses of 100% ORR and 66.7% CR rate, indicating a potential beneficial role of HSCT in improving the efficacy of CAR-T therapy. Notably, at a median follow-up of 21.5 months, nearly two-thirds of patients maintained their response until the end of follow-up.

After encountering a tumor-specific antigen *in vivo*, CARs could transmit an activation signal to T cells through the intracellular domain and cause T cell activation and expansion, which is dependent on the antigen density of tumor cells (22). For B-ALL, a dose of 10^5^/kg CAR-T cells is enough to obtain a satisfactory curative effect (23), while at least 10^6^/kg of CAR-T cells is needed in multiple myeloma (MM) and lymphoma (21, 24). Moreover, under the condition of 10^6^/kg CAR-T cell treatment, B-ALL showed significantly more severe adverse events than MM and B-cell lymphoma (20, 21, 24). In our study, 10^6^/kg of CD30 CAR-T cells failed to obtain a satisfactory response, which may be related to the special pathological characteristics of cHL. cHL is characterized by small numbers of large CD30+ Reed–Sternberg cells surrounded by a mixed infiltration of inflammatory and immune cells, which will show a low antigen density of CD30+ tumor cells. Therefore, CAR-T cell of a larger order of magnitude was tried in this study, and an exciting effect was obtained. The above-mentioned studies show that tumor load may be the key factor determining the dose level of CAR-T cell infusion, which will be further explored in the follow-up study.

PD-1 is an important negative costimulatory regulatory molecule to maintain T cell immune tolerance, and its ligand is commonly detected on cHL and NK/T lymphoma, *etc.* (8). Furthermore, upregulation of PD-1 could be seen in activated CAR-T cells (16). PD-1 blockade seems to be an ideal partner for CAR-T cell therapies. Therefore, immune checkpoint blockade in overcoming T cell exhaustion, including PD-1 gene knockout in CAR-T cells, engineering CAR-T cells to secrete anti-PD-1 and combinatorial treatment of anti-PD-1, *etc.*, is continuously tried to be applied to CD30 CAR- T cells (15, 25, 26). Studies of PD-1 blockade enhancing the eradication of tumor cells of CAR-Ts revealed the potentially critical role of the PD-1/PD-L1 pathway in CAR-T cell immunotherapy (18). Similarly, our combinatorial treatment of PD-1 inhibitor also showed an encouraging result of 100% ORR. Moreover, in four CR patients in cohort 3, all of whom were previously resistant to the checkpoint inhibitor, the combinatorial treatment of anti-PD-1 antibody was still shown to be effective. CAR-T cell expansion improvement and activity enhancement by PD-1 blockade may be the potential reasons (17, 18). The above-mentioned study suggests that the synergistic effect of PD-1 blockade seems to be unaffected by the previous PD-1 resistance status. The anti-PD-1 antibody was added with the aim of improving the expansion and reducing the exhaustion of CAR-T cells and also have the potential risk of triggering or exacerbating a CRS reaction. Acute and severe CRS mostly occurred within 2 weeks after CAR-T cell infusion. Therefore, we add anti-PD-1 antibody at 14 days after CAR-T cell infusion and continued such every 3 weeks in our study. Further optimization of the administration time of the anti-PD-1 antibody needs to be further explored.

In summary, our study suggested that PD-1 blockade yields a high clinical benefit to some extent for r/r CD30 lymphoma patients receiving anti-CD30 CAR-T therapy, and minimal toxicities were observed. These results are exciting and further support the combination of PD-1 inhibitor with anti-CD30 CAR-T therapy. In view of the small cohort in this study, larger clinical studies with more participants are required in the future to further confirm the critical role of the PD-1/PD-L1 axis in anti-CD30 CAR-T cell therapies.

## Data Availability Statement

The original contributions presented in the study are included in the article material. Further inquiries can be directed to the corresponding author.

## Ethics Statement

The studies involving human participants were reviewed and approved by the ethics committee of the Affiliated Hospital of Xuzhou Medical University and the First Affiliated Hospital of Soochow University. The patients/participants provided their written informed consent to participate in this study.

## Author Contributions

KX, CL, and WS designed the research. XW, HG, TL, DL, BZ, YZ, XS, CS, DY, DL, and ZL collected the data. XW, WS, HG, and TL analyzed and interpreted the results. XW and WS wrote the manuscript. All authors contributed to the article and approved the submitted version.

## Funding

This work was supported by the Natural Science Foundation of Jiangsu Province (BK20190985), the National Natural Science Foundation of China (81900177), and the Key Young Medical Talents of Jiangsu Province (QNRC2016791).

## Conflict of Interest

The authors declare that the research was conducted in the absence of any commercial or financial relationships that could be construed as a potential conflict of interest.

## Publisher’s Note

All claims expressed in this article are solely those of the authors and do not necessarily represent those of their affiliated organizations, or those of the publisher, the editors and the reviewers. Any product that may be evaluated in this article, or claim that may be made by its manufacturer, is not guaranteed or endorsed by the publisher.
